# 3D Indoor Positioning of UAVs with Spread Spectrum Ultrasound and Time-of-Flight Cameras

**DOI:** 10.3390/s18010089

**Published:** 2017-12-30

**Authors:** José A. Paredes, Fernando J. Álvarez, Teodoro Aguilera, José M. Villadangos

**Affiliations:** 1 Sensory System Research Group, University of Extremadura, 06006 Badajoz, Spain; fafranco@unex.es (F.J.A.); teoaguibe@unex.es (T.A.); 2 Department of Electronics, University of Alcalá, Alcalá de Henares, 28805 Madrid, Spain; jm.villadangos@uah.es

**Keywords:** acoustic positioning system (APS), unmanned aerial vehicles (UAV), time-code division multiple access (T-CDMA)

## Abstract

This work proposes the use of a hybrid acoustic and optical indoor positioning system for the accurate 3D positioning of Unmanned Aerial Vehicles (UAVs). The acoustic module of this system is based on a Time-Code Division Multiple Access (T-CDMA) scheme, where the sequential emission of five spread spectrum ultrasonic codes is performed to compute the horizontal vehicle position following a 2D multilateration procedure. The optical module is based on a Time-Of-Flight (TOF) camera that provides an initial estimation for the vehicle height. A recursive algorithm programmed on an external computer is then proposed to refine the estimated position. Experimental results show that the proposed system can increase the accuracy of a solely acoustic system by 70–80% in terms of positioning mean square error.

## 1. Introduction

The production of Unmanned Aerial Vehicles (UAVs), commonly known as drones, has experienced a notable increase in the last few years, with the development of new applications both in the civil and the military market. As is well known, the autonomous operation of these airships outdoors depends on their Global Navigation Satellite System (GNSS) receiver, which provides them with location coordinates in a global geographical system. However, the accuracy of GNSS is insufficient for many applications, and even worse, GNSS signals are severely attenuated indoors where they cannot be reliably used. For these reasons, many researchers are focusing their attention on the development of a solution to the accurate and reliable location of UAVs in indoor environments. This development includes the exploration of alternative technologies that must be compared in terms of coverage, accuracy, precision, cost and adaptability to existing infrastructures. One of these technologies is ultra-wideband (UWB) radar [1], which provides centimeter accuracy, strong multipath resistance and a good material penetration capability, which can be useful under Non-Line-of-Sight (NLoS) conditions, thanks to its very large bandwidth. However, this technology cannot be considered an ideal solution, since the transmission of information can be adversely affected under strong scattering conditions. Laser technology has also been proposed to conduct Simultaneous Localization and Mapping (SLAM) tasks in the design of a fully-autonomous UAV [2], but its financial costs remain relatively high. A different approach is based on the exploitation of existing infrastructures and the so-called Signals-Of-Opportunity (SOP) to conduct a scalable positioning at a reduced cost. Most of these systems rely on radio-frequency technology, as the one described in [3], which proposes the combined use of the GSM network with a Received Signal Strength (RSS) fingerprinting to improve the 50–200 m range accuracy provided by this mobile network. The RSS measurement is also employed with the WLAN networks extensively deployed in buildings, factories and malls. Most commercial UAVs incorporate a video camera that can be used for positioning and mapping purposes by performing a 3D scanning of the environment and a pattern recognition procedure. This use can be extended to unknown environments through image processing and the comparison with databases [4]. Furthermore, [5] proposes the use of a passive depth camera, mounted on a UAV with an ad hoc setup, specially designed for outdoor scene applications. According to the author’s experiments, this system can be adequate for mapping projects with lower resolution and accuracy requirements. A similar approach appears in [6], where the authors utilize a stereo vision sensor as an indoor positioning system for UAVs. The system makes use of two video cameras for stereo vision capture and a set of fast algorithms to provide position information with reliable accuracy in real time. The possibilities of this technology are wide, with admissible implementation costs both at the hardware and the software level, but its characteristic computation times are still long for real-time use.

All the technologies described above can be used in a complementary way to achieve a more robust design, taking advantage of the strongest points of each one depending on the task to be performed. They can also be combined with an Inertial Measurement Unit (IMU) to improve the accuracy of the positioning and state parameters. For instance, the authors in [7] use it with a monocamera and a laser range finder, and data from a structured light scanner, UWB and an inertial navigation system is integrated in [8]. Furthermore, a navigation system stabilizing the LiDAR measurement plane using an IMU sensor is proposed in [9]. Finally, a fusion of monocular vision with laser measurements, IMU and altimeter is presented in [10].

This work proposes the combination of spread spectrum ultrasound and Time-Of-Flight (TOF) cameras as new technologies in the field of indoor UAV positioning. In the first case, in spite of being considered a classical technology for the local positioning community, with well-recognized characteristics such as high precision and reduced cost, the use of ultrasound for the location and navigation of UAVs is still very early in development. In the second case, TOF cameras are a relatively new technology that has begun finding application in indoor positioning very recently.

This work is thus a first attempt to combine all the knowledge acquired by the authors in the field of general purpose Ultrasonic Local Positioning Systems (ULPS) and their most recent experiences in the use of TOF cameras, with the aim of designing an accurate 3D indoor positioning system for UAVs. To properly contextualize this effort, the next section first presents a review of general purpose ULPS, from the first narrowband proposals appearing in the late 1990s, to the most advanced spread spectrum systems developed recently. This section concludes with a review of recently-proposed range imaging positioning systems. Next, Section 3 presents the proposed system based on the combination of these two technologies. Some experimental results obtained with this system in a real scenario are provided in Section 4, and finally, the main conclusions of this work are drawn in Section 5.

## 2. Ultrasonic and Range Imaging Indoor Positioning

### 2.1. Ultrasonic Positioning

As stated before, active ultrasonics has been extensively used in the design of indoor positioning systems, and it is today considered a classical solution to this technological challenge [11,12]. First, general-purpose ULPS, dating from the late 1990s and early 2000s, made use of envelope detection techniques to estimate the Time-Of-Arrival (TOA) of constant-frequency narrowband ultrasonic pulses. This is, for example, the case of the Active Bat system [13,14], where wireless badges (bats) carried by personnel or attached to certain equipment emit 40-kHz ultrasonic pulses of a 50-μs duration after being triggered over a wireless link. These pulses are received by a set of ceiling-mounted sensors that measure the pulse TOAs and compute the badge 3D position by spherical lateration. A different approach is proposed in the Constellation System [15], where a set of ultrasonic emitters is deployed at known locations in the environment. These beacons emit a 40-kHz ultrasonic pulse after receiving an infrared trigger code from the unit to be located, which communicates with the beacons one-at-a-time. The receiving unit needs to compute at least three TOAs from the emissions of different beacons to obtain its position by spherical lateration. Note that, in this case, the device to be located is in charge of computing its own position using the signals emitted from different beacons. This architecture has been defined by some authors as privacy-oriented, in contrast to the centralized architecture of the Active Bat system. A third example of a narrowband ULPS that cannot be considered centralized nor privacy-oriented is the Cricket system [16,17]. This system is based on a set of independent beacons that incorporate an RF transceiver, an ultrasonic emitter and an ultrasonic receiver. Each beacon can compute its own position by measuring the TOAs of the 40-kHz ultrasonic pulses of 125 μs in duration emitted by nearby beacons, which also broadcast their own position through a 433-MHz RF signal. Again, spherical lateration is used to compute the desired location.

All the systems described above featured very simple emitter and receiver acoustic modules, but at the expense of providing a limited positioning accuracy of some decimeters with also high sensitivity to in-band noise. A solution to these limitations is provided by the pulse compression technique extensively used in radar systems [18]. As is well known from this theory, the precision of a range measurement can be improved if the effective bandwidth of the received signal is increased while keeping its energy constant. This bandwidth extension can be achieved by shortening the duration of a constant frequency pulse, but then, its amplitude should be increased above the limit imposed by the linear operation regime of real amplifiers and transducers. An alternative is to modulate the original waveform and use a matched filter to detect it at the receiver. This spread spectrum technique had been successfully incorporated in the development of high precision airborne sonars some years before [19,20,21], so its application in the field of ULPS was just a question of time. One of the first broadband ULPS is presented in [22,23], where the authors propose the use of 511-bit Gold codes to modulate a 50-kHz ultrasonic carrier with a bit period of 50 μs, thus giving a total emission duration of 25.55 ms. In [22], eight receivers are installed in the ceiling of an office room to configure a centralized architecture that measures the TOAs of the signals emitted by a set of synchronized transmitters. The authors reported positioning accuracies slightly above 2 cm when using spherical lateration in a noisy environment. In [23], a privacy-oriented architecture is presented with positioning accuracies around 5 cm. A similar privacy-oriented architecture is presented in [24], based on the modulation of a 50-kHz carrier with 127-bit Gold codes and a bit period of 20 μs, for a significantly shorter emission duration of 2.54 ms.

However, the notably improved precision and robustness to noise of broadband ULPS do not come for free. The use of longer and simultaneous emissions gives rise to new problems that may hinder the detection of these signals. Some of the most recent works in the field of broadband ULPS are focused on the proposal of solutions for some of these problems, such as Doppler shift [25], multipath propagation [26] or Multiple Access Interference (MAI) [27]. A parallel trend is the development of ULPS for commercial portable devices, whose limited hardware resources and computing capability require a complete redesign of the signal processing algorithms [28,29]. The current state of evolution of these systems suggests their use for the accurate positioning of UAVs, a technological challenge that must face new problems that were not present in the positioning of previous receivers, such as the effect of the rotors on the propagation channel.

### 2.2. Range Imaging Positioning

There are only a few works that propose the use of range imaging systems to perform positioning tasks, probably because of the need for an initial calibration step and the high computational cost of image processing. In [30,31], the authors present an algorithm to identify the room where the camera is installed, via object detection and its comparison with a specific building database. No precise location inside the room is provided, though. In [32,33], depth information is used to detect walls and other vertical plane furniture. Furthermore, in [34], depth information is combined with odometry and laser ranging to improve the autonomous navigation of a robot. All these works rely on a mobile positioning system to cover relatively large areas, and their use is limited by the battery life. In [35], the authors present a method for real-time people tracking and coarse pose recognition in a smart room, using ceiling-mounted and downward-pointed TOF cameras. The number of cameras needed is the main limitation of this proposal. A similar people detection and tracking system is presented in [36].

Although range imaging does not seem to be the most suitable technology to perform the real-time location of fast-moving vehicles, this work proposes the use of a TOF camera to complement the positioning information provided by a central ultrasonic system. The details of this complementary operation are described in the next section.

## 3. Proposed Indoor Positioning System

**Figure 1:**
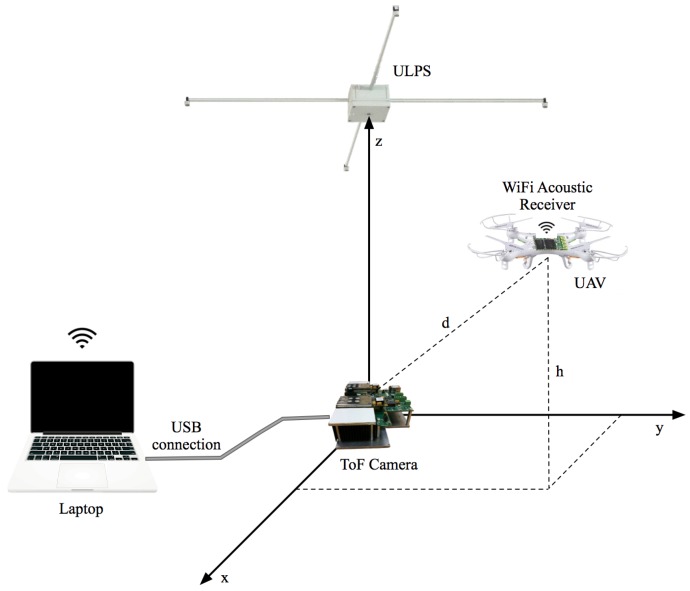
General view of the proposed system. ULPS, Ultrasonic Local Positioning System.

Figure 1 depicts a diagram of the proposed system, which is composed of three different elements. The central element is an ultrasonic emitter module installed in the ceiling of the room. The acoustic signals emitted by this system are acquired by a portable receiver module installed in the drone top cover. Below the emitter module and at the floor level, there is a TOF-camera pointing upwards. The third element is an external computer that collects both the acoustic signal acquired by the portable receiver and the range-image generated by the TOF camera, which are used to compute the UAV location. All these modules are described next.

### 3.1. Ultrasonic Module

The ultrasonic emitter module consists of five transducers or beacons, distributed around a 70.7 cm × 70.7 cm square structure. The coverage area is of 30 m${}^{2}$ when it is installed at the ceiling at a height of 2.8 m. Five different 63-bit Kasami sequences [37] have been used to accurately identify every beacon of the ULPS. The codes have been Binary Phase Shift Keying (BPSK) modulated with two periods of a sinusoidal carrier at 41.67 kHz, in order to adapt their spectral features to the frequency response of the ultrasonic transducers (Prowave 328ST160 [38]). The modulated codes have been previously generated offline and sampled at 500 kHz, so 12 samples per carrier period are considered.

If $K=k_{m}\left[l\right]\in \left\{-1,1\right\};\;0\leq m\leq M-1;\;0\leq l\leq L-1$ is a family of Kasami codes of length L=63, in which the M=5 codes with better correlation properties are selected for encoding the beacons of the ULPS and s[n] is the modulation symbol with $N_{c}=2$ carrier cycles and oversampling factor equal to $O_{f}=f_{s}/f_{c}=500\;\mathrm{kHz}/41.67\;\mathrm{kHz}=12$, the modulated pattern to be emitted can be represented as (1):

(1) $${\mathrm{sk}}_{m}\left[n\right]=\sum_{i=0}^{L-1}k_{m}\left[i\right]\cdot s\left[n-i\cdot N_{c}\cdot O_{f}\right]$$

**Figure 2:**
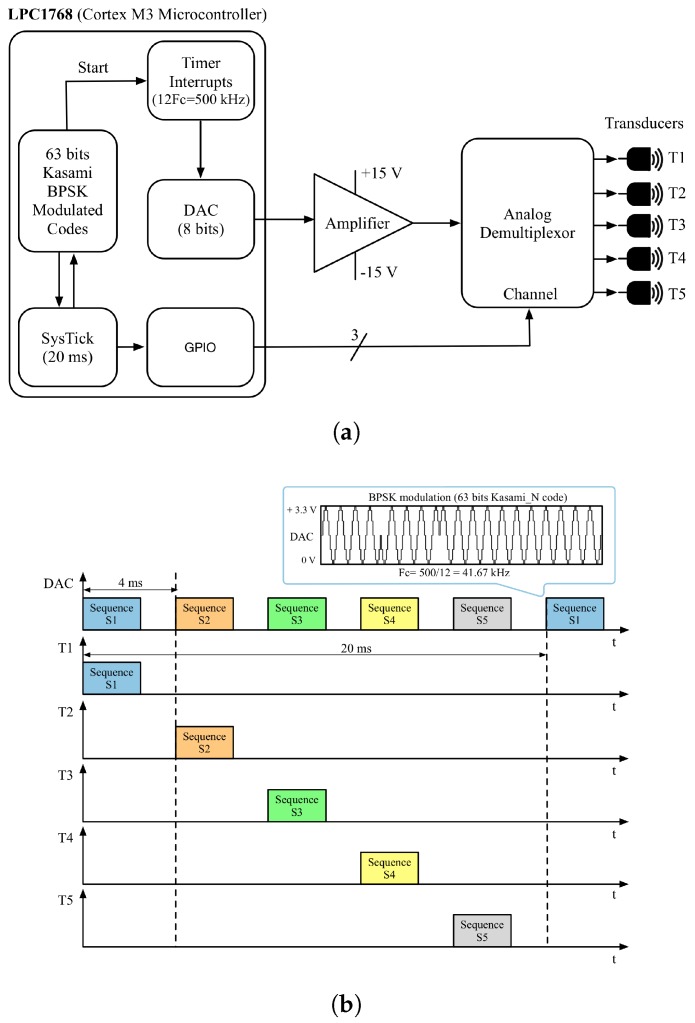
Ultrasonic emitter module, (**a**) Block diagram of the ultrasonic emitter module, (**b**) Beacons’ emission pattern.

The modulated codes are stored in the memory of the ULPS microcontroller that manages all of the emission process. All parameters regarding the code design (type of code, length and oversampling factor) can be easily configured according to the demands of the final application. The emitter module is based in a Cortex-M3 microcontroller. Since only one Digital to Analog Converter (DAC) is available in this microcontroller, a Time Division Multiple Access (TDMA) technique has been implemented, which also reduces multipath and MAI. Figure 2a shows the block diagram of this module, composed of an LPC1768 microcontroller, an amplifier to adapt the transducers’ voltage level and a multiplexer to select among the different Kasami-modulated sequences. Furthermore, Figure 2b shows the DAC output signal, where the BPSK modulation and the TDMA emission scheme can be observed. Note the 4-ms delay between consecutive emissions and the total emission cycle of 20 ms.

**Figure 3:**
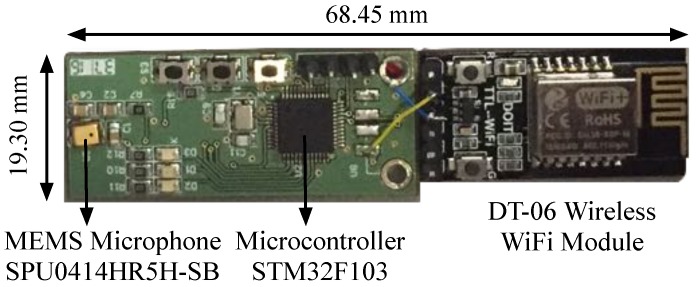
Ultrasonic receiver module.

These signals are acquired by a specifically-designed receiver module. This portable module is based on low cost components and includes an MEMS SPU0414HR5H-SB microphone by Knowles Acoustic (Itasca, IL, USA) [39] with an adequate bandwidth at 41.67 kHz and a serial WiFi module for data wireless transmissions. This module, represented in Figure 3, has a total weight of about 6 g, which is far below the drone payload.

The signals captured by the microphone are high-pass filtered and amplified by a configurable gain amplifier. A STM32F103CBT6 microcontroller by STMicroelectronics (Geneva, Switzerland) [40] digitizes the incoming ultrasonic signals at a sampling rate of 100 kHz. Note that a decimation by five has been included to reduce the amount of data to be processed. The receiver buffer size ensures the reception of one complete emission from all five beacons. A buffer with 2000 samples (4 ms) of the ultrasonic signal is periodically acquired and sent via a UART interface to a serial WiFi module (DT-06 Wireless WiFi) [41] from whence these samples are transmitted to the external process unit.

**Figure 4:**
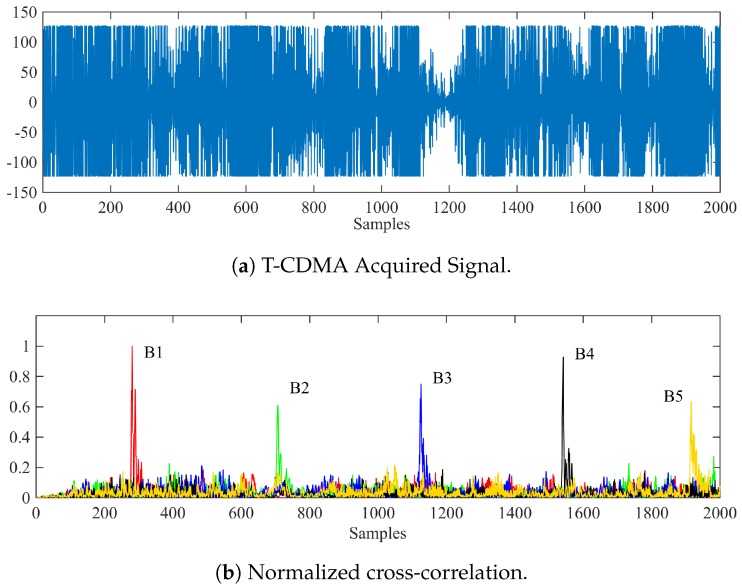
(**a**) Acquired signal for the receiver and (**b**) normalized cross-correlation with a set of five Kasami codes assigned to the beacons.

In the external unit, the acquired signal is first cross-correlated with all Kasami patterns to obtain the set of correlation peaks that are represented in Figure 4. From these peaks and taking into account the 4-ms delay between consecutive emissions, the Time Differences Of Arrival (TDOAs) can be easily extracted to compute the receiver’s location by multilateration. This technique has been previously used in the literature, as can be seen in [28,42].

The ultrasonic system described in this section has been demonstrated to provide an accurate positioning of the receiver in a horizontal plane whose height is known a priori, for instance in [43]. However, the performance of this system is significantly poorer when used to provide a 3D positioning with a variable height, due to the particular distribution of beacons in the ceiling plane and the consequences of this distribution on the Vertical Dilution of Precision (VDOP). For this reason, this work proposes the use of a TOF camera as a means to provide an initial estimation of the receiver’s UAV height that will be later refined by means of an iterative geometrical correction based on the 2D coordinates obtained from a 2D multilateration procedure. The operation of this camera and the positioning algorithm are described in detail in Section 3.3.

### 3.2. ToF Camera

**Figure 5:**
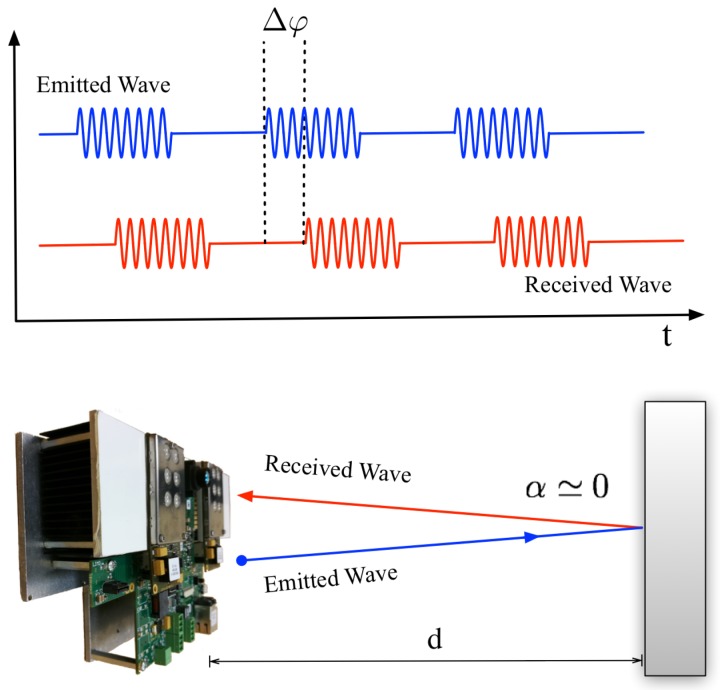
TOF camera working principle. At the top, a temporal diagram representing the phase shift between the emitted and the received wave. At the bottom, a scene diagram: the angle between incident and reflected waves is near zero because the distance *d* is much greater than the separation between the LEDs (emitters) and the lens (receiver).

As mentioned above, the second element of the proposed system is a TOF camera installed at the origin of coordinates to get depth information of the scene. Every pixel, built in CCD/CMOS technology [44], works independently. The working principle (Figure 5) consists of emitting continuously an infrared wave, but modulated by a square signal. The lens receives the returning wave in a matrix of smart pixels. They are able to compute information about this optical signal: the constant DC offset, the modulation amplitude and the phase shift, from which the distance between the camera and the measured object is calculated as,

(2) $$d=\frac{c}{2}\times \frac{\Delta \varphi }{2\pi f}$$

where c is the speed of light and *f* is the modulation signal frequency. It is important to take into account that the angle between incident and reflected waves on the target is near zero because the distance *d* is much greater than the separation between the LEDs (emitters) and the lens (receiver). A detailed mathematical explanation of this behavior can be found in [45], and a clarifying study from the point of view of the CCD/CMOS pixels is included in [46]. Hence, this kind of camera provides not only 2D information in a single image, but also information about the third dimension. Since they do not provide exact volume data, these devices are also known as 2.5D cameras.

Moreover, this device must be properly calibrated in order to get absolute information. Many works in the literature deal with this crucial topic. These works try to elaborate different algorithms and situations to obtain intrinsic and extrinsic parameters that correct certain errors, mainly due to the lenses [47,48,49]. This procedure is usually arduous and time-consuming. The idea in this paper is to avoid this initial calibration and consider only the radial distance to the drone with a slight correction due to radial distortion, since depths measured far from the vertical axis suffer from small deviations. This effect can be easily corrected by adding an offset from the axis to the last pixel, taking into account how far this point is.

**Figure 6:**
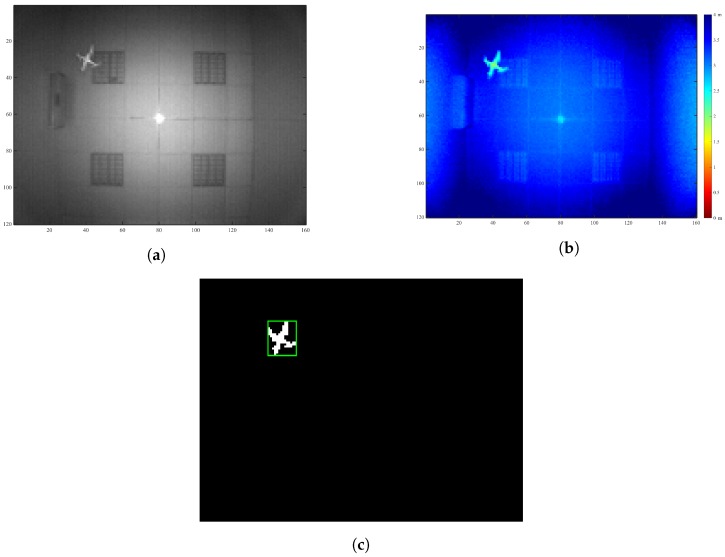
Images from the TOF camera. The configuration has been chosen so as to reach all possible distances in a proper way, as can be appreciated. In this case, the drone flew approximately 1.9 m from the lens. (**a**) Image of amplitudes, (**b**) Image of distances, (**c**) Drone recognition after subtracting the background.

There are nowadays different solutions in the TOF camera market to address this problem. In our case, a prototype board from Bluetechnix has been used to have as many different configurations available as possible. The kit—by Bluetechnix Lab GmbH (Wien, Austria)—is composed mainly of a PMD PhotonICs 19k-S3 TOF 3D chip and a Spartan 6 FPGA for real-time image processing [50]. The illumination module provides the scene with 850-nm wavelength light (12 LEDs), not visible to the human-eye. Related to the variable settings, f=10 MHz has been chosen and an integration time (time during which the pixels are capturing light; the equivalent of exposure time in RGB cameras) of 2.5 ms. This configuration allows one to obtain a good enough image so as to process it. Figure 6 shows an example of images taken from the camera. Figure 6a shows the returning wave amplitude in each pixel, and in Figure 6b, different colors represent different depths, as can be read from the vertical color bar. In this snapshot, in which the drone was flying around pixels $\left(x_{p},y_{p}\right)=\left(40,30\right)$, the measured range is about 1.9 m.

This depth image is sent to the external computer, where a simple image recognition procedure starts. Previously, a series of preceding images had been averaged in order to get the background *B* in the instant *t*:

(3) $$B\left(x,y,t\right)=\frac{1}{N}\sum_{i=1}^{N}D\left(x,y,t-i\right)$$

where *D* is a depth image (different in each time) and (*x*, *y*) are the image pixels. *N* is the number of measurements (100 for this study). Now, the foreground F(x,y,t) can be achieved via subtracting the background from every instant frame:

(4) F(x,y,t)=D(x,y,t)−B(x,y,t)

so that all elements greater than a threshold (maximum of 1 cm in this system) correspond to new objects appearing in the scene. Then, F(x,y,t) can be converted into a binary image (Figure 6c), where white pixels build new objects. After that, the connected elements are identified making use of MATLAB’s Image Processing Toolbox, separating them from each other. Thus, the largest connected region will be the drone. Additionally, the range value *d* is taken from the geometric centroid of this area.

**Figure 7:**
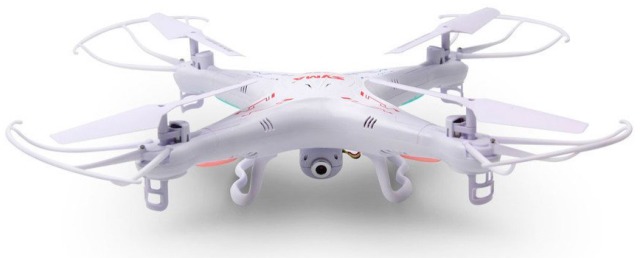
Image of the SYMA X5C quadcopter drone.

This section is finally completed with a brief description of the selected drone, depicted in Figure 7. This is a low cost device, with a mass of 107 g and a size of 31cm×31cm×8cm. The drone area visible from the camera is approximately 28 cm × 28 cm, since the propellers are not detected due to their fast rotation.

### 3.3. Positioning Strategy

**Figure 8:**
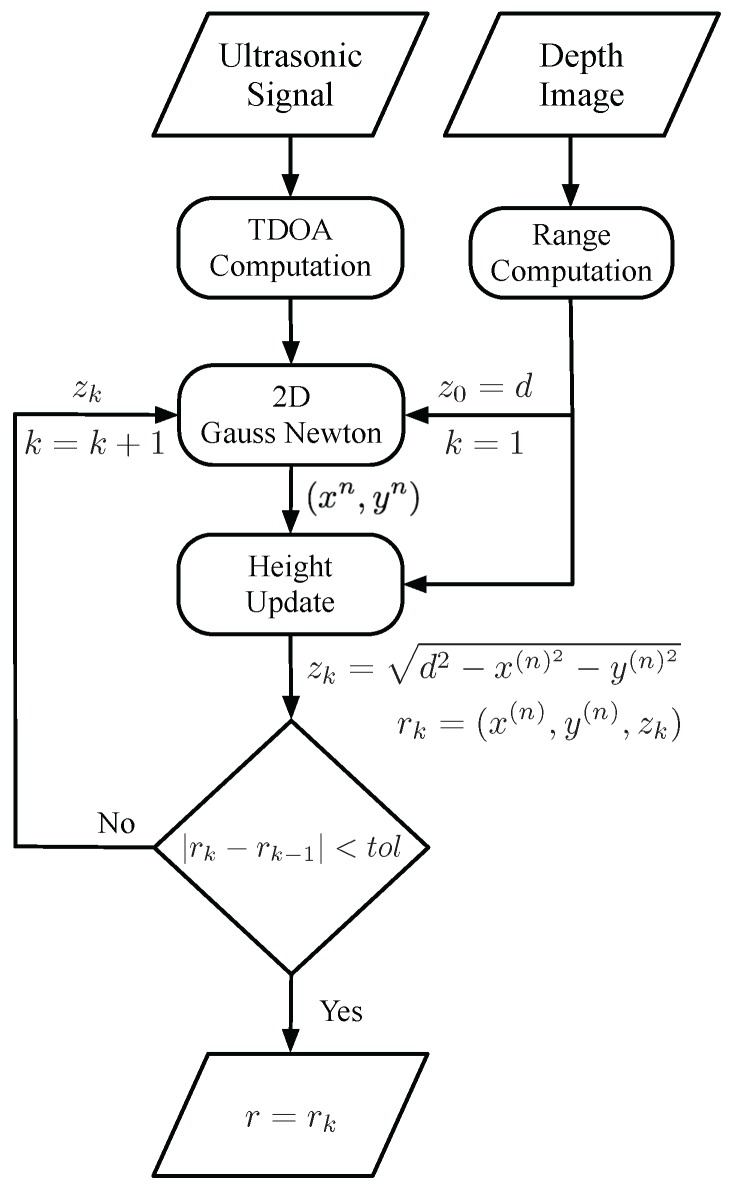
Flux diagram of the positioning algorithm.

The proposed positioning algorithm executed in the external computer appearing in Figure 1 can be better explained with the help of the flux diagram represented in Figure 8. As mentioned before, this computer receives the ultrasonic signal from the portable receiver installed in the drone and the depth image from the TOF camera. It then computes the TDOAs of the ultrasonic emissions and the range to the drone, respectively, following the procedures already described in the two previous sections. All this information is used as the input of a two-Dimensional Gauss–Newton (2D-GN) algorithm that iteratively solves the horizontal position of the drone using the range as an estimate for the drone’s height, i.e., updating the drone’s position r=(x,y,z) as:

(5) $$r^{\left(n\right)}=r^{\left(n-1\right)}+{\left(A^{T}\times A\right)}^{-1}A^{T}\times B$$

where $r^{\left(0\right)}=\left(0,0,d\right)$, and matrices *A* and *B* are given by:

(6) $$A=\left[\begin{array}{ccc} \frac{x_{2}-x^{\left(n-1\right)}}{d_{2}^{\left(n-1\right)}}-\frac{x_{1}-x^{\left(n-1\right)}}{d_{1}^{\left(n-1\right)}} & \frac{y_{2}-y^{\left(n-1\right)}}{d_{2}^{\left(n-1\right)}}-\frac{y_{1}-y^{\left(n-1\right)}}{d_{1}^{\left(n-1\right)}} & \frac{z_{2}-z}{d_{2}^{\left(n-1\right)}}-\frac{z_{1}-z}{d_{1}^{\left(n-1\right)}}\\ \frac{x_{3}-x^{\left(n-1\right)}}{d_{3}^{\left(n-1\right)}}-\frac{x_{1}-x^{\left(n-1\right)}}{d_{1}^{\left(n-1\right)}} & \frac{y_{3}-y^{\left(n-1\right)}}{d_{3}^{\left(n-1\right)}}-\frac{y_{1}-y^{\left(n-1\right)}}{d_{1}^{\left(n-1\right)}} & \frac{z_{3}-z}{d_{3}^{\left(n-1\right)}}-\frac{z_{1}-z}{d_{1}^{\left(n-1\right)}}\\ \frac{x_{4}-x^{\left(n-1\right)}}{d_{4}^{\left(n-1\right)}}-\frac{x_{1}-x^{\left(n-1\right)}}{d_{1}^{\left(n-1\right)}} & \frac{y_{4}-y^{\left(n-1\right)}}{d_{4}^{\left(n-1\right)}}-\frac{y_{1}-y^{\left(n-1\right)}}{d_{1}^{\left(n-1\right)}} & \frac{z_{4}-z}{d_{4}^{\left(n-1\right)}}-\frac{z_{1}-z}{d_{1}^{\left(n-1\right)}}\\ \frac{x_{5}-x^{\left(n-1\right)}}{d_{5}^{\left(n-1\right)}}-\frac{x_{1}-x^{\left(n-1\right)}}{d_{1}^{\left(n-1\right)}} & \frac{y_{5}-y^{\left(n-1\right)}}{d_{5}^{\left(n-1\right)}}-\frac{y_{1}-y^{\left(n-1\right)}}{d_{1}^{\left(n-1\right)}} & \frac{z_{5}-z}{d_{5}^{\left(n-1\right)}}-\frac{z_{1}-z}{d_{1}^{\left(n-1\right)}} \end{array}\right]$$

(7) $$B=\left[\begin{array}{c} \left(d_{2}^{\left(n-1\right)}-d_{1}^{\left(n-1\right)}\right)-\left(d_{2}-d_{1}\right)\\ \left(d_{3}^{\left(n-1\right)}-d_{1}^{\left(n-1\right)}\right)-\left(d_{3}-d_{1}\right)\\ \left(d_{4}^{\left(n-1\right)}-d_{1}^{\left(n-1\right)}\right)-\left(d_{4}-d_{1}\right)\\ \left(d_{5}^{\left(n-1\right)}-d_{1}^{\left(n-1\right)}\right)-\left(d_{5}-d_{1}\right) \end{array}\right]$$

$\left(x_{i},y_{i},z_{i}\right)$ being the position of the *i*-th beacon, $d_{i}^{\left(n-1\right)}$ the distance from beacon *i* to the drone’s (n−1)th position estimate and $d_{i}$ the experimental distance measured to beacon *i*. Note that in the two-dimensional algorithm, the *z* coordinate appearing in matrix *A* keeps its original value. Once the 2D-GN algorithm has provided an estimate for the drone’s horizontal position, this information is used to update the drone’s height *z* using trigonometry as follows:

(8) $$z=\sqrt{d^{2}-{x^{\left(n\right)}}^{2}-{y^{\left(n\right)}}^{2}}$$

Finally, if the updated position $r=(x^{\left(n\right)},y^{\left(n\right)},z)$ changes above a certain margin of tolerance, the updated height is fed back to the 2D-GN algorithm to obtain a more accurate horizontal position. In other cases, the updated position is provided as the final estimate.

## 4. Experimental Results

**Figure 9:**
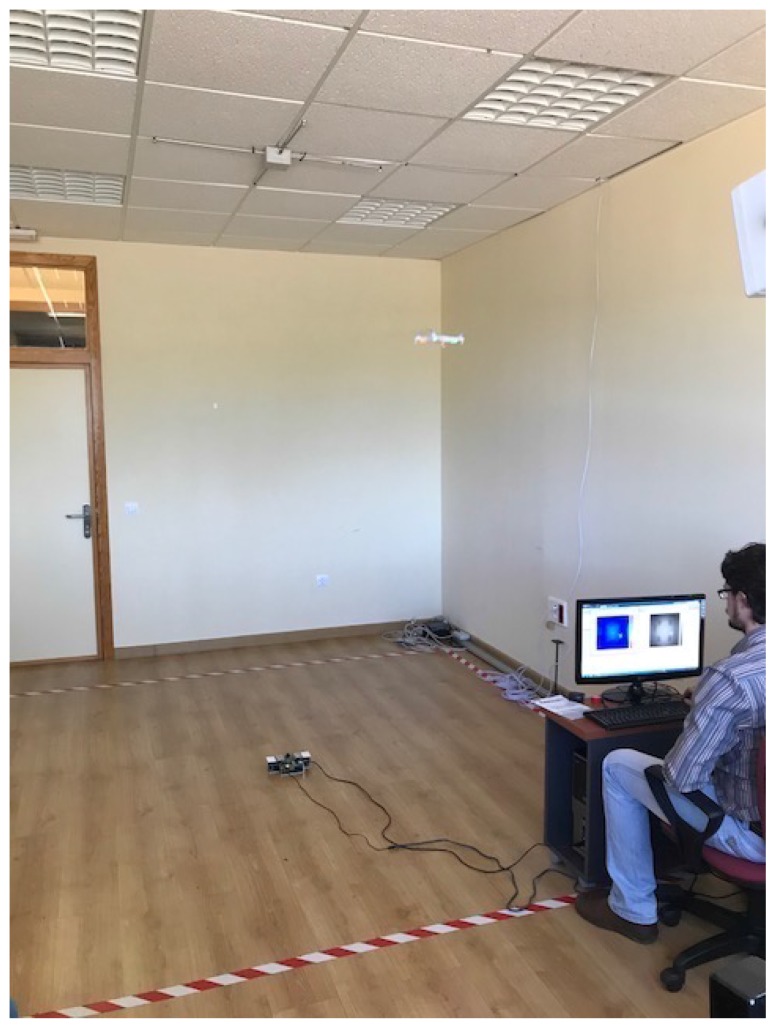
Snapshot of the experimental site, with the ULPS installed in the ceiling of an office room and the TOF-camera placed at the origin of coordinates.

The main objective of this work is to hybridize the ultrasonic and optical technologies to develop an accurate 3D positioning system for UAVs. Figure 9 shows a picture of the actual experimental site, where the performance of the proposed positioning system has been analyzed by defining three different test points: p1=(0,0,0.97) m, p2=(0.60,−0.37,1.59) and p3=(0.60,−0.90,1.41) and computing a total of 100 positions in each of these points.

First of all, a comparison between the proposed hybrid system and a solely ultrasonic system performing 3D multilateration has been conducted. Table 1 shows the results of this comparison in terms of the positioning Mean Squared Error (MSE) and its corresponding Standard Deviation (SD). As can be seen, the proposed system performs significantly better in all the test positions, with relative improvements between 70% and 80% in the MSE. An important decrease in the SD is also observed, which also indicates an improvement in the system precision. This table can be compared with the results provided in [1], where a UWB system is developed to localize a flying drone. In this work, errors less than 0.176 m for 50% of cases are reported.

**Figure 10:**
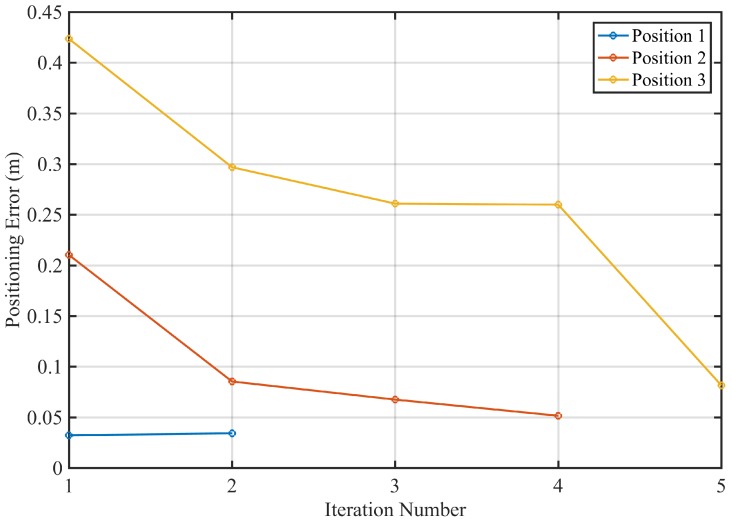
Number of necessary algorithm iterations to converge to a solution.

Since the proposed positioning strategy is based on an iterative multilateration procedure, it is also important to study the rate of convergence of this process. Figure 10 represents the MSE against the number or iterations in the three tests points. As expected, the number of required iterations increases with the distance from the drone to the vertical *z*-axis, since the TOF-camera provides an initial estimation of this drone’s height as its range to the origin of coordinates. In any case, a maximum number of five iterations is required to obtain a stable estimation in the furthest point, with a refreshing time of less than one second.

Finally, it is important to remark that the system-measured update rate is about 0.42 s, which is good enough to allow real-time operation in a constrained environment where the drone operation velocity must be limited.

## 5. Conclusions

This work has proposed the use of a hybrid broadband ultrasonic and optical positioning system to perform the accurate positioning of UAV in indoor environments. The proposed acoustic module is based on a T-CDMA scheme, where the sequential emission of five spread spectrum ultrasonic codes is performed to estimate the vehicle horizontal position following a 2D multilateration procedure. The signals emitted by this module are acquired by a portable receiver attached to the top cover of the UAV. This receiver is based on a MEMS microphone, a microcontroller and a WiFi module for wireless data transmission to an external computer. The optical module is based on a TOF-camera that sends a range-image of the scene to the same computer. This computer first uses this image to obtain an initial estimation of the UAV height, which is combined with the ultrasonic TDOAs to refine the 3D position following an iterative procedure. Experimental results obtained in a real environment confirm a significant improvement in the performance of this system with respect to that of the system that only uses the acoustic module to perform 3D multilateration. These results also show that the proposed iterative algorithm needs a maximum of five iterations to converge to a stable solution.

## Figures and Tables

**Table 1:** Mean Squared Error (MSE) and its Standard Deviation (SD) in each position under study.

(m)	Position 1		Position 2		Position 3	
**Acoustic System**	0.140	(0.089)	0.350	(0.032)	0.303	(0.257)
**Hybrid System**	0.037	(0.009)	0.067	(0.017)	0.081	(0.061)
	MSE	SD	MSE	SD	MSE	SD
